# Alternative to Conventional Solutions in the Development of Membranes and Hydrogen Evolution Electrocatalysts for Application in Proton Exchange Membrane Water Electrolysis: A Review

**DOI:** 10.3390/ma16186319

**Published:** 2023-09-20

**Authors:** Klara Perović, Silvia Morović, Ante Jukić, Krešimir Košutić

**Affiliations:** Faculty of Chemical Engineering and Technology, University of Zagreb, Marulićev trg 19, 10000 Zagreb, Croatia; smorovic@fkit.unizg.hr (S.M.); ajukic@fkit.unizg.hr (A.J.)

**Keywords:** proton exchange membrane water electrolysis (PEMWE), green hydrogen, renewable energy, electrocatalysts, noble metals, non-noble metals

## Abstract

Proton exchange membrane water electrolysis (PEMWE) represents promising technology for the generation of high-purity hydrogen using electricity generated from renewable energy sources (solar and wind). Currently, benchmark catalysts for hydrogen evolution reactions in PEMWE are highly dispersed carbon-supported Pt-based materials. In order for this technology to be used on a large scale and be market competitive, it is highly desirable to better understand its performance and reduce the production costs associated with the use of expensive noble metal cathodes. The development of non-noble metal cathodes poses a major challenge for scientists, as their electrocatalytic activity still does not exceed the performance of the benchmark carbon-supported Pt. Therefore, many published works deal with the use of platinum group materials, but in reduced quantities (below 0.5 mg cm^−2^). These Pd-, Ru-, and Rh-based electrodes are highly efficient in hydrogen production and have the potential for large-scale application. Nevertheless, great progress is needed in the field of water electrolysis to improve the activity and stability of the developed catalysts, especially in the context of industrial applications. Therefore, the aim of this review is to present all the process features related to the hydrogen evolution mechanism in water electrolysis, with a focus on PEMWE, and to provide an outlook on recently developed novel electrocatalysts that could be used as cathode materials in PEMWE in the future. Non-noble metal options consisting of transition metal sulfides, phosphides, and carbides, as well as alternatives with reduced noble metals content, will be presented in detail. In addition, the paper provides a brief overview of the application of PEMWE systems at the European level and related initiatives that promote green hydrogen production.

## 1. Introduction

Excessive consumption of fossil fuels must be replaced with renewable ones, especially because the worldwide power demand will reach 24 or 26 TW by 2040, with emitted CO_2_ emissions of 37–44 GT per year by 2040 [1]. Among the various fuel sources, hydrogen is often referred to as the “fuel of the future”, with a high energy density of 140 MJ kg^−1^, which is more than twice that of conventional solid fuels (50 MJ kg^−1^) [2]. Other benefits of promoting the hydrogen economy include energy security by reducing oil imports, less pollution and better urban airy quality, sustainability by taking advantage of renewable energy sources, and economic viability by potentially shaping future global energy markets [3].

Currently, total global hydrogen production is about 500 billion cubic meters (bcm), with the produced hydrogen being extensively used in petroleum refining processes, in the petrochemical and chemical industries, in fuel cells, and in the fertilizer industry. Most of the hydrogen (about 96%) is produced from non-renewable fossil fuels, mostly by steam reforming of methane. Therefore, the hydrogen produced in this way is characterized by a lower purity and a high concentration of harmful greenhouse gases [2]. Only 4% of the hydrogen produced worldwide is produced in a renewable way by water electrolysis, which is a promising way of producing high-purity hydrogen without carbon emissions [1].

Synergies between hydrogen, electricity, and renewable energy sources are urgently needed [4]. Over the past decades, the increasing prices of electricity have postponed and hindered the production of electrolytic hydrogen, but this low percentage of its application is expected to increase with recent growth in energy capacity based on renewable sources like wind turbines and photovoltaics [5]. This is also supported by the revised Renewable Energy Directive 2018/2001/EU [6], which has established a new binding target for renewable energy in the EU for 2030 of at least 32%.

Proton exchange membrane electrolysis (PEMWE) is considered the most promising form of hydrogen production based on high efficiencies and suitable current densities even at moderate temperatures [4]. Combined with renewable energy sources, PEM electrolyzers can produce electrolytic hydrogen that can work as an energy vector/carrier and energy storage medium and thus overcome the intermittency of typical renewable energy sources [5]. After being stored, the hydrogen produced by electrolysis can be converted back into electricity when needed or used to refill fuel-cell-based cars [4].

Highly dispersed carbon-supported Pt- and Ir-based materials are currently being benchmark catalysts for the hydrogen evolution reaction (HER) and oxygen evolution reaction (OER) in PEMWE, but practical development to satisfy growing demand requires the use of cheaper catalysts [5,7]. The capital costs that currently make this technology less attractive could be lowered by reducing loading and/or substituting the expensive noble materials used for the fabrication of catalyst layers and hardware [5]. Furthermore, this article focuses on an overview of recent novel electrocatalysts that represent promising cathode materials for hydrogen production by PEM water electrolysis, considering non-noble metal alternatives together with reduced noble catalysts loading. The general mechanism of hydrogen evolution in water electrolysis, the specific performance of PEM water electrolyzers, and a summary of the novel electrocatalyst materials used with substituted and reduced noble metal contents are summarized.

## 2. Hydrogen Economy in Europe

Hydrogen represents the cornerstone of clean energy production; therefore, the European Commission proposed a Hydrogen Strategy for a Climate Neutral Europe in 2020 [8] in order to increase hydrogen supply and demand as well as to make the widespread production of hydrogen possible in 2050. Likewise, the strategy sets targets for the installation of at least 40 GW of renewable hydrogen electrolyzers by 2030. Furthermore, the European Commission has put together the “Fit for 55” packages, which relate to the EU’s plan to reduce greenhouse gas emissions by at least 55% from 1990 levels by 2030, as required by European Climate Law [9,10]. The European Commission’s proposed REPowerEU plan [11] aims to rapidly reduce the European Union’s dependence on Russian fuel by setting a target of 10 million tons of renewable hydrogen produced domestically annually with an additional 10 million tons of hydrogen imported annually by 2030. To achieve these goals, the EU must rapidly increase the production of electrolyzers. According to industry estimates, to achieve the target of 10 million tons of renewable hydrogen produced in the EU, an electrolyzer capacity of 90–100 GW_LHV_ should be installed [12]. The electrolyzer manufacturing capacity should be scaled up significantly since the current capacity of electrolyzer manufacturers in Europe is estimated at 1.75 GW_LHV_ per year [12]. The development of innovative low-carbon technologies such as electrolyzers is supported by the EU Innovation Fund, which focuses on projects that can lead to significant emissions reductions. Hydrogen project promotors can also be supported by the European Investment Bank (EIB) [13] which, in recent years, has financed EUR 550 million in hydrogen projects.

Focusing exclusively on the application of PEM water electrolysis and the fulfillment of the European 2030 goals, the current operational state of PEM water electrolyzers and the goals that need to be achieved (Table 1) are given as part of the *Strategic Research and Innovation Agenda 2021–2027* published by the Clean Hydrogen Partnership in 2022 [14]. The set goals are primarily related to the reduction in capital and operating costs which will enable the achievement of market competitiveness of the mentioned technology for industrial application.

**Figure 1:**
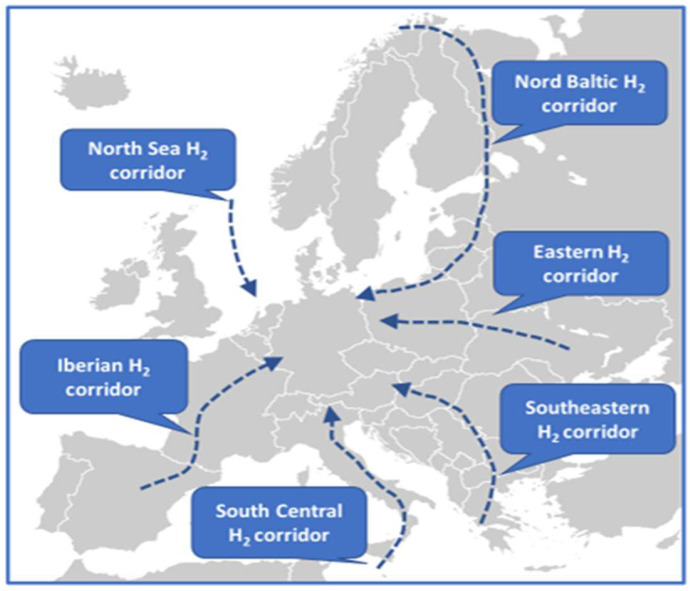
Hydrogen supply corridors in Europe. Modified from [16].

Apart from the proposed plans [10,11], the European Commission has prepared a Hydrogen Public Funding Compass [15] to guide stakeholders to access information on the most important public funding programs and funds for renewable and low-carbon hydrogen. To accelerate Europe’s 2030 hydrogen goals, potential hydrogen supply corridors are envisioned, including South Central Europe, the Iberian Peninsula, the North Sea, the Nordic-Baltic region, and the Eastern, and Southeastern H_2_ supplying corridors (Figure 1) [16].

At the European Union level, there are currently several alliances and organizations promoting the development of the hydrogen economy. Examples include the European Clean Hydrogen Alliance [17], Hydrogen Europe [18], and Clean Hydrogen Joint Undertaking (Clean Hydrogen Partnership) [19]. In January 2023, the Clean Hydrogen Partnership, the successor of Fuel Cells and Hydrogen Joint Undertaking (FCH JU), selected nine hydrogen valley projects with a total funding of EUR 105.4 million [20]. Hydrogen valleys are regional ecosystems that demonstrate how hydrogen technologies work synergistically, but also complementarily, with other elements such as electricity grid, batteries, renewable energy production, gas infrastructure, etc.

As already mentioned, the rapid development of water electrolyzers and the intensification of scientific research in this field are closely related to the European Commission’s intention to reduce energy dependence on Russia by expanding electrolyzer production capacity in the EU. In line with EU policy, electrolyzer manufacturers in Europe have agreed to increase their production capacities tenfold to 17.5 GW per year [21]. Table 2 provides a list of the main companies involved in the production of PEM electrolyzers in the EU.

## 3. Proton Exchange Membrane for Water Electrolysis (PEMWE)

The first water electrolyzer based on the concept of a solid polymer electrolyte was idealized by Thomas Grubb at General Electric in the 1960s, using a solid sulfonated polystyrene membrane as the electrolyte. This term also refers to proton exchange membrane or polymer electrolyte membrane (both with the acronym PEM) water electrolysis and, less commonly, to solid polymer electrolyte (SPE) water electrolysis [5]. The first PEM electrolysis journal was published in 1973 by Russell and co-workers [23] at General Electric, who used a PEM electrolysis cell to produce hydrogen from water splitting. It is interesting to note that at that time, the authors proposed the idea of using hydrogen as an energy storage device for off-peak periods in the power grid, as well as the possibility for its distribution and use for automotives. Nowadays, these are the main drivers for PEM electrolysis technology [24].

Water electrolysis technologies are divided into three categories depending on the electrolyte used: alkaline water electrolysis (AWE), PEMWE, and solid oxide water electrolysis (SOWE) [25]. PEM systems offer several advantages over the other two electrolysis technologies, such as higher hydrogen production rates, more compact design, and higher energy efficiency. Compared to alkaline electrolysis, which usually uses potassium hydroxide (KOH) solution as the electrolyte, the solid electrolyte membrane (thickness 50–250 μm) of the PEM electrolyzer significantly reduces gas crossover, allowing operation at high pressures [26].

In terms of commercial availability, AWE is emerging as the main competitor to PEM technology. A later section describes in more detail the commercial production and the use of PEM electrolyzers in the EU. Table 3 shows the basic differences between the mentioned technologies (AWE, PEMWE and SOWE) as well as the basic advantages and disadvantages of PEM technology in relation to the selected alkaline water electrolysis. It is also important to emphasize that, recently, more attention has been devoted to the electrolysis of seawater instead of scarce freshwater in the production of clean hydrogen. Although the mentioned technology is not the focus of this paper and will not be discussed in detail, it is important to mention that PEM electrolyzers and cells with a liquid electrolyte are considered the most suitable for the production of hydrogen from seawater electrolysis [27]. The most commonly used electrocatalysts for HER in seawater are various noble-metal-based materials, including noble-metal-based chalcogenides with cation vacancies, graphene-supported noble-metal-containing alloys, etc. [28].

The production of so-called green hydrogen by water electrolysis with the use of renewable electricity costs on average two to three times more than the production of so-called blue hydrogen obtained from natural gas by steam reforming. If the electricity input is added, the electrolyzer itself is the second largest cost component [22].

Compared to AWEs for hydrogen production, PEM electrolyzers are characterized by higher efficiency and current densities, as well as stable surrounding hydration conditions due to constant membrane exposure to the liquid phase of water, which makes the electrolytic membrane fully hydrated. There are several drawbacks that slow down the widespread application of this technology. First, a PEM water electrolyzer operating at pressures up to 70 bar can produce electrolytic-grade hydrogen and oxygen with high efficiency. As the pressure increases, the concentrations of hydrogen and oxygen produced can reach critical levels, increasing the risk of explosive gas mixture formation. To avoid this, the gas transfer of the generated gases must be reduced. The use of chemically and mechanically robust PFSA membranes can also be improved, considering their degradation, aging, and susceptibility to contamination.

Furthermore, commercialization of PEMWE technology requires testing of PEMWE cells under real (more aggressive) working conditions, since most of the current PEM water electrolyzers have been tested in the laboratory under stationary conditions (temperature, current density, and pressure). All this leads to the conclusion that large-scale implementation of PEMWE systems is hindered by high investment costs as well as the dependence on noble metal catalysts.

For these reasons, in the continuation of this paper, a detailed review of the scientific achievements and advances in the field of development of modified membranes and cathode materials with high application potential for hydrogen production by PEM water electrolysis is given, with the aim of presenting suitable materials that could affect initial investment cost reduction, as well as making this technology market competitive.

### 3.1. Components of PEMWE

**Figure 2:**
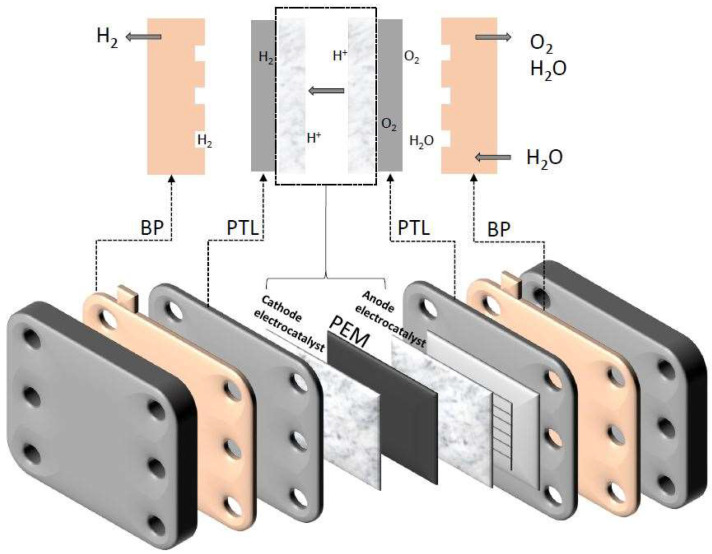
Key components of the PEMWE system. Modified from [30].

The PEMWE system consists of a membrane electrode assembly, which actually consists of an integrated proton exchange membrane (PEM), an anode and cathode electrocatalyst, and a porous transport layer (PTL), also referred to as a gas diffusion layer (GDL) in the literature. The catalysts can be deposited directly on the membrane or on the PTL. Flow field plates or bipolar plates (BPs) serve as separation plates and allow heat, charge, and mass transfer (Figure 2) [30].

In the PEMWE system, water is supplied to the anode side of the cell, flows through the channels of BP and PTL, and reaches the anode catalyst layer. At the anode, the water molecules dissociate into O_2_, protons, and electrons. O_2_ evolves from the system, and electrons and protons pass through the membrane to the cathode catalyst layer, where H_2_ is produced. The hydrogen formation reaction can proceed via two mechanisms: the Volmer–Heyrovsky mechanism, where protons are first adsorbed on the surface of the metal electrode, where M–H* is formed (Volmer step). In the Heyrovsky step, M–H* reacts with H^+^ ions or electrons to form H_2_. In contrast to the Volmer–Heyrovsky mechanism, in the Volmer–Tafel mechanism, M–H* can react with another M–H*, also forming H_2_ [31].

There are various hydrogen storage systems that can be divided into physically based and material-based technologies. Physically based technologies include compressed gas, liquid, and cryocompressed forms [32]. The hydrogen produced by the reduction in protons on the cathode side must be stored in special vessels. To facilitate storage, it is recommended to produce H_2_ at an elevated pressure and to use high-pressure electrolysis. High-pressure electrolysis has been successfully applied in practice, and several commercial systems operate at pressures up to 20 bar [33]. Further increasing the pressure to several hundred bar allows direct storage of the generated hydrogen in pressure vessels, but also requires overcoming certain difficulties. The biggest problem caused by working under high pressure is the so-called phenomenon of gas crossover, which occurs during electrolysis in the entire area of the proton exchange membrane, i.e., hydrogen and oxygen generated on both sides of the membrane could enter the membrane space and mix with each other, increasing the risk of gas explosion [34]. Besides all the advantages of physically based technologies for hydrogen storage, there are other disadvantages related to storage capacity, energy consumption, requirements for the vessels to withstand high pressures, and high costs, as well as safety issues related to leakage, bursting, and fire hazards. Due to the numerous drawbacks of physically based technologies, material-based (chemical) hydrogen storage technologies have been developed that offer a number of advantages, including higher hydrogen storage capacity at room temperature, lower hydrogen storage pressure, and slower hydrogen release rate. Metal hydrides, for example, have the potential to store both hydrogen and thermal energy. However, complex thermal management systems, expensive catalysts, and stability issues remain drawbacks, so most industries continue to rely on physically based hydrogen storage technologies [32,35].

#### 3.1.1. Porous Transport Layers (PTLs)

PTLs are a porous medium located between PEM and BPs. They are used for liquid/gas transport, i.e., transport of water to the catalyst layer, where generation of O_2_, H^+^, and electrons takes place, and transfer of O_2_/H_2_ to the separator plates. Considering the functions of such a porous medium, the PTL material must meet certain requirements such as corrosion resistance, good electron conductivity, and good mechanical strength. Due to the highly acidic conditions, high overpotential, and the presence of O_2_, the range of materials for producing such porous media is limited to metals or carbon materials [30].

Titanium and stainless steel are most commonly used for PTLs fabrication due to their high corrosion resistance [5,36]. Moreover, carbon materials can also be used for the fabrication of PTLs, but only for the fabrication of the cathode PTL, as the high oxidation potential of the anode would severely affect its mechanical strength. On the other hand, titanium is susceptible to passivation, which primarily leads to deterioration of durability and the formation of an oxide layer, which then affects the contact between PTLs and the fluid collector and also affects the conductivity of the medium. In addition to passivation, hydrogen embrittlement can also occur [30]. In the study by Rakousky et al., uncoated Ti–PTL was examined to determine its rate of degradation. It was found that the highest rate of degradation (194 μV h^−1^) occurred at constant operation at 2 A cm^−2^ and was attributed to the voltage increase [37]. Hydrogen embrittlement and passivation can be prevented by coating titanium with noble metals, but this greatly affects the cost of the entire system. In their study, Kang et al. showed that novel gold (Au)-sputtered titanium thin/tunable liquid/gas diffusion layers (TT-LGDL) improved interfacial contacts and reduced ohmic and activation losses through their advantages of flat surface and thin structures [38]. Furthermore, Liu et al. showed that no degradation of the electrolyzer occurred due to the formation of a <10 nm thick IrOx layer on the iridium-coated PTL. It was shown that the Ir coating was oxidized, and the TiOx layer under the iridium was not further passivated, unlike the unprotected PTL, which may improve durability and reduce the cost of today’s expensive PTLs, i.e., a balance should be found between capital cost and durability [39].

In general, Ti mesh, Ti felts, Ti foams, and sintered Ti powders are commonly used in PEMWE systems for the production of anode PTL [40,41]. On the other hand, the use of materials such as mesh, foam, etc., results in random pore size and pore size distribution. For this reason, researchers have focused on optimizing the structure, i.e., pore size and distribution, as well as the thickness of the porous medium. It was shown that the optimal pore size of PTL is between 10 and 13 μm [34], while the optimal porosity is about 30%. Therefore, it is important that the pores in the medium are not too large, as this reduces the efficiency of electron transport; the pores also cannot be too small, as the removal of the generated gas in the system is hindered, i.e., the transfer resistance increases.

#### 3.1.2. Bipolar Plates (BPs)

BPs are multifunctional components whose main functions are to ensure the flow of reactants and products, the electrical connection of adjacent cells in the so-called stack, and mass and heat transport. Considering the harsh environment in PEMWE, the materials used for the production of BPs must have high mechanical strength, corrosion resistance, high conductivity, impermeability, and low cost [42].

The most commonly used materials for manufacturing BP are graphite, stainless steel, and titanium. Although graphite has high electrical conductivity, it has not proven to be a good option for PEMWE systems due to its low mechanical strength, susceptibility to corrosion, high cost, and the fact that it cannot be used for BP anodes but only for cathodes due to oxidation of the carbon surface. Considering that BP accounts for almost 50% of the total cost, 80% of the weight, and 50% of the volume of the entire PEMWE system, intensive work is being done to find suitable substitute materials and optimize the geometry [31,43,44].

Materials such as stainless steel, titanium, and their alloys have been shown to be good substitutes for graphite. However, as in the case of PTLs, titanium has excellent properties such as high mechanical strength and corrosion resistance, but is subject to passivation, i.e., the formation of an oxide layer. Therefore, coatings and alloys have been used as a solution for the protection of titanium and stainless steel plates. Titanium plates can be coated with noble metals to overcome deficiencies and achieve satisfactory durability and performance. For example, gold (Au) coatings can be applied to protect the titanium surface from oxidation under severe corrosion conditions. In the study by Jung et al., the stress degradation rate of the Au-coated titanium-based bipolar plate was shown to be five times lower than that of the conventional carbon bipolar plate [45]. However, coating with noble metals is very expensive from the perspective of large-scale electrolytic cells. Therefore, to reduce the cost, it is necessary to focus on the production of low-cost materials with satisfactory properties. Wakayama et al. investigated titanium (Ti) bipolar plates coated with titanium suboxide (Ti_4_O_7_) as a substitute for Pt-coated titanium bipolar plates as a cost-effective way to fabricate the PEM water electrolysis system. In their study, Ti_4_O_7_-sputtered Ti was shown to have a very low contact resistance (4–5 mΩ cm^−2^) before and after voltage application, which is equivalent to that of gold or platinum coatings [46]. Moreover, in the study by Rojas et al., the multilayer Ti/TiN coating on SS 321 showed the best performance with 0.02% weight loss, current at 2 V_SHE_ to 436 μA cm^−2^, and interfacial contact resistance after corrosion test of up to 9.9 mΩ cm^−2^, indicating good protective properties of the coating [47].

In addition to optimizing the composition, optimizing the geometry of the flow channel also proved effective in increasing corrosion resistance and ensuring uniform flow distribution of reactants and products on the surface area of the field plate. Therefore, it is very important that the flow field plate ensures uniform distribution of reactants across the catalytic reaction surface in order to provide a way for collection of products and a conductive path to the reaction site. In the study by Toghyani et al., five flow field patterns were compared: parallel, one-way serpentine, two-way serpentine, three-way serpentine, and four-way serpentine to determine the best performance in terms of distribution of the molar fraction of hydrogen produced, current density, temperature, and pressure drop. It was found that the two-way serpentine provides the best performance for PEM electrolyzers due to relatively higher hydrogen production rate, more uniform temperature distribution, and reasonable pressure drop [48].

##### PFSA–PEM Membranes

A thin perfluorosulfonic acid (PFSA) membrane, known by the trade name Nafion^®^, is most commonly used as a solid electrolyte in PEMWE systems. Nafion^®^ is a fluoropolymer of sulfonated polytetrafluoroethylene first commercialized by DuPont (Wilmington, DE, USA) in the mid-1970s. It is commercially available in thicknesses of approximatively 100 μm [49]. Nafion^®^ consists of a neutral semi-crystalline polymer backbone (polytetrafluoroethylene-PTFE) and a randomly tethered side chain of polysulfonyl fluoride vinyl ether with the ionic group ${\mathrm{SO}}_{3}^{-}$ linked to a specific counterion. Considering the length of its side chain, Nafion^®^ can be described as “long-side chain” (LSC) polymer. The nature of the covalently bonded side chain and backbone result in phase separation, which is further enhanced by the addition of solvent molecules (water). Due to its phase-separated morphology, Nafion^®^ has demonstrated excellent ion and solvent transport capabilities. Moreover, the main advantages of Nafion^®^ are also its high proton conductivity and water permeability, as well as its high chemical and mechanical resistance. Despite its exceptional properties, there are certain disadvantages, which are mainly reflected in its high price, high disposal costs due to the presence of fluorine in the material skeleton, and reduced proton conductivity under high temperature conditions (>100 °C) [50,51].

Currently, state-of-the-art commercially available membranes, in addition to Nafion^®^, are Flemion^®^ (Asahi Glass, Tokyo, Japan) and Aciplex^®^ (Asahi Kasei, Tokyo, Japan), which are polymers with identical structures to Nafion^®^, but with shorter side chains. Although Flemion^®^ and Aciplex^®^ have good properties, they are chemically less stable than Nafion^®^. In the 1980s, Dow Chemical Company (Midland, MI, USA) introduced a perfluorinated ionomer with a short side chain (SSC-Dow Membrane) without a fluoroether group in the side chain, containing only two -CF_2_ groups. In addition, Solvay Specialty Polymers (Brussels, Belgium) launched HyflonR Ion (known as Aquivion^®^). During the same period, the 3M™ Corporation (Maplewood, MN, USA) developed an ionomer (the 3M™ ionomer) with a fluoroether-free side chain containing four -CF_2_ groups (the structures are given in Table 4). In addition, GORE—SELECT, a reinforced composite membrane, was introduced by W. L. Gore & Associates (Newark, DE, USA) by incorporating a strong hydrophobic reinforcement layer into a PFSA ionomer that improves dimensional stability in response to hydration [50,52].

There are several criteria for high efficiency of PEM membranes, such as high proton conductivity, low gas crossover capability, good thermal properties, low swelling ratio, sufficient water absorption, and high mechanical and chemical resistance [58,59]. In order to overcome the shortcomings of PFSA membranes and thus improve membrane performance, various modification strategies have been developed to address the decomposition and performance issues. To improve structure, stability, and performance, the side chain chemistry of PFSA can be modified by doping or chain modification itself, or the membrane can serve as a host for reinforcements and additives. Ionomers can be impregnated or doped with radical scavengers (to reduce radical formation), with inert hydrophobic mechanical support layers (to improve mechanical properties and dimensional stability under humidity fluctuations), with inorganic particles (to improve the stability of the membrane itself), or with hygroscopic fillers (to improve water retention inside the membranes at high temperatures).

In general, modifications can be divided into two categories: (1) reinforcement with another polymer such as expanded polytetrafluroethylene (ePTFA) or blending with other polymers by electrospinning; and (2) impregnation of the ionomer with additives, metal salts or inorganic dopants [50].

The incorporation of a reinforcing layer, e.g., a mesh made of ePTFA, has proven effective. Besides ePTFA, there are other porous reinforced materials such as poly (vinylidene fluoride) (PVDF) electrospinning microporous membrane, polyvinyl alcohol (PVA) microporous membrane, etc. The porosity of this hydrophobic mesh allows the use of membranes with smaller thicknesses and smaller EW ionomers that would otherwise severely compromise mechanical stability. The microporous support layer is incorporated in the center of the ionomer, where it is filled with ionomer, which contributes to the formation of a continuous proton transport channel and water transport throughout the thickness of the membrane. The consequences of membrane reinforcement can affect dimensional anisotropy. Reinforced membranes exhibit better in-plane stability, which means less in-plane swelling. The reinforced structure increases yield strength and elastic modulus and decreases in-plane swelling. Lower in-plane swelling is therefore critical for reducing swelling-induced mechanical stress and increases resistance to defect propagation. In addition, reinforced membranes have been shown to be less sensitive to humidity fluctuations. Examples of commercially available membranes include Gore Select membranes, Nafion XL, and HP, which are PFSA-reinforced membranes with ePTFE. Along with porous reinforcement materials, impregnation of PFSA ionomers with electrospun nanofibers such as poly(phenylsulfone) (PPSU), poly(acrylic acid) (PAA), or poly(ethylene oxide) (PEO) and reinforcing particles such as carbon nanotubes can be used to improve mechanical properties and water retention [50,60,61,62].

Impregnation of the ionomer with additives, metal salts, or inorganic dopants is used to improve membrane performance, e.g., dimensional stability, thermomechanical stability, or conductivity at lower humidity and higher temperatures. The most common fillers used to improve membrane performance are metal salts, hygroscopic inorganic fillers such as SiO_2_ [63,64], ZrO_2_ [65,66], TiO_2_ [66,67], functionalized inorganic fillers, and particles such as carbon nanotubes [68]. Composite membranes with metal oxides (SiO_2_, TiO_2_, or WO_2_) showed promising properties for use at high temperatures. In their research, Baglio et al. and Antonucci et al. studied the operation of Nafion–TiO_2_ and Nafion–SiO_2_ composite membranes at elevated temperatures (>100 °C) [69,70]. Both studies showed that due to the presence of inorganic hygroscopic fillers in the polymer mass, the performance at elevated temperatures exhibited better water retention and more uniform distribution of water in the composite membrane, reducing ohmic resistance and improving electrolytic performance [63,66].

Although great progress has been made in reducing the cost and eliminating the deficiencies of PFSA membranes, there is still a great need to improve their chemical and mechanical properties and to reduce their degradation, aging, or contamination while improving important properties such as proton conductivity.

##### Hydrocarbon-Based Membranes

Hydrocarbon-based membranes show potential for application in PEMWE systems. They showed good thermal stability, similar proton conductivity, and the production cost is lower compared to PFSA [52]. The application potential was particularly evident for hydrocarbon-based membranes fabricated from sulfonated derivatives such as sulfonated poly(phenylene sulfone)—sPPS, sulfonated poly(arylene ether sulfone)—sPAES, sulfonated polysulfone—sPSf, sulfonated poly(ether ether ketone)—sPEEK, and sulfonated polybenzimidazole—sPBI. Although the use of alternative membranes offers many advantages, a careful evaluation of their mechanical, chemical, and thermal stability and durability is very important before they are finally installed in PEMWE systems, where the systems must be in operation for more than 50,000 h [31].

In sPPS membranes, the aromatic ring bearing the sulfonic acid group is connected to two sulfone bonds (-SO_2_^−^) that are strongly electron-withdrawing (electron acceptor), i.e., the sulfonic acid groups are directly bonded to a strongly electron-withdrawing poly(phenylene sulfone) backbone [71]. This structural feature is the reason for the significantly higher acidity of sPPS compared to other sulfonated hydrocarbons, resulting in improved proton conductivity, especially at low relative humidity [72]. Furthermore, sPPS membranes showed good thermal stability and low gas crossover capability [31]. In the sPEEK membrane, the phenyl ring is linked to ether bonds and carbonyl groups, while the sulfonic acid group is linked to the phenyl ring [73]. The properties of the membranes largely depend on the degree of sulfonation (DS), whereby higher DS always favors excellent proton conductivity [74]. The potential for application is also evident in its low-cost fabrication and thermal, chemical, and mechanical stability [31]. Moreover, sPAES is widely used for PEM applications due to its simple and inexpensive production and modification as well as its excellent membrane properties. In addition, due to its excellent mechanical properties, thinner membranes can be produced, which helps to reduce ohmic resistance. However, the properties of hydrocarbon-based membranes, including SPAES, are also affected by the DS of the polymers [75]. The overview of recently reported PEM membranes (PFSA and hydrocarbon-based) in PEMWE systems is given in Table 5.

Hydrogen produced by electrolysis has the lowest emission rate, but its production cost is nevertheless the highest due to high capital and electricity costs. The Energy Transitions Commission (ETC) assumes that the cost of hydrogen in Europe today is 5.10 EUR/kg (assuming USD 780/kW capital cost) [76]. The price of hydrogen produced by the PEMWE system depends not only on the price of electricity, but also on the efficiency of the cell used and its lifetime, i.e., materials, catalysts, electrolytes (membranes), as well as working pressures and temperatures [77]. For example, Areva H_2_Gen has shown that the price of hydrogen produced with PEMWE is USD 3.90/kg for 1 MW PEMWE (8000 operating hours per year) at an electricity price of about USD 55/MWh. International Energy Agency’s (IEA) predicts that green hydrogen will cost about USD 1–2.50/kg by 2050 [78]. In order to reduce the price of hydrogen produced, it is necessary to invest mainly in PEMWE systems powered exclusively by renewable energy sources. In addition, the efficiency of electrolyzers must be improved by introducing new materials, focusing on low-cost, durable, high-performance materials such as durable and more active catalysts, thinner membranes, and less critical raw materials [77]. Table 6 contains PEMWE’s key performance indicators related to advances in membrane materials and catalysts. Comparing Table 6 with Table 5, Table 7, and Table 8, it is clear that with respect to 2022, some of the targeted parameters are being improved by the introduction of new materials. However, the new materials implemented in PEMWE have not yet reached commercial levels, and further research is needed in order to achieve the 2050 targets. Furthermore, a review of PEMWE performance data reported in the literature shows that performance results vary so widely that it is difficult to draw conclusions about technological improvements and research and development directions. It is crucial that testing protocols be standardized to allow comparison of performance evaluations between different studies [79].

**Table 5:** Brief overview of recent reports on PEMWE with PFSA- and hydrocarbon-based membranes.

Membrane	Material	Thickness/μm	Cathode Loading /Cathode Catalyst	IEC	Conductivity/mS cm^−1^	T/°C	Current Density	Stability Test	Ref.
PFSA									
Fumapem^®^/graphene	Per-Fluorinated Sulfonic Acid (PFSA)/PTFE copolymer; 0.38 *w*/*v*—graphene loading	112	-	0.82 mmol g^−1^	115	80	-	-	[80]
(S-TiO_2_)/Nafion	sulfated titania (S-TiO_2_)-dopped Nafion	100–110	0.5 ± 0.1 mg cm^−2^ of Pt Pt/Vulcan XC-72—30%	0.82 ± 0.01 meq g^−1^	≈70	100	4 A cm^−2^ at 2 V	-	[81]
biaxially stretched Nafion 117	PFSA, Nafion series 117	28.2 ± 1.7	0.4 mg cm^−2^ of Pt Pt/C—0.5	0.92 meq g^−1^	*σ*_i_ = 73 *σ*_t_ = 54	80	3 A cm^−2^ at 1.9 V.	0.4 A cm^−2^ for 50 h	[82]
hBN/Nafion	monolayer hexagonal boron nitride/Nafion	-	0.4 mg cm^−2^ of Pt	-	18.7 ± 0.9	70	-	0.4 A cm^−2^ for 100 h (50°C)	[83]
Aq830-PSU(5 wt %)	electrospun polysulfone fiber web/Aquivion^®^	45 ± 2	0.5 mg cm^−2^ of Pt and 33 wt% Nafion^®^ ionomer (5 wt% solution) Pt/C—40 wt%	-	220	80	2 A cm^−2^ at 1.76 V	-	[84]
3M 729/ePTFE (annealed at 180°) AQ 720/ePTFE (annealed at 180°)	ePTFE porous support was impregnated with 3M 729 and AQ 720 and annealed at different temperatures	55–60	0.25–0.30 mg cm^−2^ of Pt Pt/C—40 wt%	1.30 meq g^−1^ 1.31 meq g^−1^	106 112	80	-	-	[62]
NPP-95	Nafion/poly(acrylic acid)/poly(vinyl alcohol) 95:2.5:2.5	50–60	0.1 mg cm^−2^ of Pt	0.84 meq g^−1^	189.2 ± 12.1	80	4.310 A cm^−2^ at 2.0 V	-	[59]
Hydrocarbon membranes									
BPSH50 (random)	hydrocarbon-based sulfonated poly(arylene ether sulfone)	40–50	0.5 mg cm^−2^ of Pt Pt/C—0.4	1.86 meq g^−1^	178	80	5.3 A cm^−2^ at 1.9 V	3 A cm^−2^ for 90 h	[85]
CSPPSU	crosslinked sulfonated polyphenylsulfone	70–130	0.3 mg cm^−2^ of Pt Pt/C—20 wt%	1.71 meq g^−1^	30	150	0.456 A cm^−2^ at 1.8 V	-	[86]
sPPS	sulfonated poly(phenylene sulfone)	115 ± 12	0.5 mg cm^−2^ of Pt Pt/C—1.6 wt%	2.78 meq g^−1^	-	80	3.48 ± 0.03 A cm^−2^ at 1.8 V	1 A cm^−2^ for 80 h	[87]
SPAES50	Sulfonated poly(arylene ether sulfone)	20	0.4 mg cm^−2^ of Pt with a 10 wt% P50 content Pt/C –40 wt%	1.89 meq g^−1^	330.1 ± 6.0	90	1.069 A cm^−2^ at 1.6 V	-	[75]
12%MKT-NW/C-sPEEK	MXene/potassium titanate nanowire cross-linked sulfonated polyether ether ketone	-	-	1.88 meq g^−1^	9.7	room temperature	-	-	[88]
4%MXene-Cu_2_O/sPEEK	Titanium carbide-copper oxide cross-linked sulfonated poly ether ether ketone	-	-	1.66 meq g^−1^	10.5	30	-	-	[89]
SPPNBP_3 SPPNBP_5	multi-block copolymer membranes consisting of sulfonated poly(p-phenylene) and naphthalene containing poly(arylene ether ketone)	42–57	0.5 mg cm^−2^ of Pt Pt/C—0.4	2.05 2.49 meq g^−1^	200 152	80	4.8 5.5 A cm^−2^ at 1.9 V	-	[90]
G-sPSS-1.95	grafting a highly sulfonated poly-(phenylene sulfide sulfone) side chain onto a poly(arylene ether sulfone) main chain	50–60	0.4 mg cm^−2^ of Pt Pt/C—40 wt%	1.95 meq g^−1^	290	90	6 A cm^−2^ at 1.9 V	1 A cm^−2^ for 50 h	[91]

## 4. Electrocatalysts for Hydrogen Production

The question of using expensive materials for the fabrication of the electrodes in PEMWE technology dates back to the 1960s when the first PEMWE devices were developed. Regarding the empirical aspects, these early systems were considerably efficient, presenting performances of 1.88 V @ 1 A cm^−2^ or 2.24 V @ 2 A cm^−2^, with a cell life of over 15,000 h without substantial performance degradation. Back then, the question concerning the high cost of used catalysts was also raised, and for these systems, catalyst layers were based on Ir and Pt black with high metal loading [5]. This work will put aside the analysis of anode electrocatalysts, and the focus will be on cathode ones.

Harsh electrochemical environments (high anodic overpotential, low pH, the presence of strong oxidants, possibility of operating at higher temperatures) require the use of precious metal compounds as electrocatalysts in PEMWE [92]. For HER, highly dispersed carbon-supported Pt-based materials, with low overpotential close to zero and a Tafel slope around 30 mV/decade, are currently benchmark catalysts, but the practical developments to satisfy the growing demands require the use of cheaper electrocatalysts [7]. The cathode catalyst represents a considerable portion of the total system cost, especially if degradation or corrosion of the carbon support occurs [5]. Nowadays, cathode side metal loading is maintained at approximately 0.5–1 mg cm^−2^, and further decreases will be needed for values reaching below 0.2 mg cm^−2^ [2]. The PEMWE with the non-noble cathodes exhibited the current density of 0.35–0.73 A cm^−2^ at 2.0 V in the operating temperature range of 80–90 °C, which were still lower than that with noble Pt/C cathodes (1.46–2.71 A cm^−2^) [93].

Typically, electrocatalysts are chosen based on their specific characteristics such as particle size, pore structure, good electrical conductivity with high surface area, and corrosion stability under oxidizing conditions [94]. For better electrochemical performance and maximum consumption of catalyst surface, the electrocatalysts are usually supported on the carbon because of its superior electrical conductivity, mechanical and thermal stability, large surface area, environmental friendliness, and relatively low cost [94]. Different types of carbons are studied for the application in PEMWE, such as carbon black (CB), carbon nanomaterials (CNMs), graphene, fullerenes, carbon nanotubes (CNTs), and heteroatom-doped CNMs(N-CNTs) [94].

There are different proposed ways of decreasing Pt loading in PEMWE, but most of the solutions require a finding of cheaper non-noble catalyst, the use of specific supports to achieve better dispersion and a higher catalytic surface, and the formation of noble metals alloys by addition of new elements to the main compound [95].

### 4.1. HER Electrocatalysts with Substituted Noble Metals Content

According to the volcano-plot theory, the electrocatalytic activity is controlled by the H adsorption free energy for HER, and only noble metals can efficiently electrolyze the HER [7]. But, during the operation of PEMWE, conventional HER catalysts suffer fewer kinetic and stability problems due to relatively facile reduction in protons in acidic media and the negative potential window for operation [96]. This opens the possibility of using non-noble metals as HER catalysts for PEMWE. But, pure transition metals, such as Ni (−0.280 V_SHE_), Co (−0.277 V_SHE_), Fe (−0.440 V_SHE_), and Mo (−0.200 V_SHE_) are also less stable in PEMWE electrolysis because they can undergo dissolution during HER because of the values of their standard reduction potentials [4,96]. The use of these transition metals could be improved by composite formation with other promising materials since the main requirements within this field are the improvement of the surface area of the electrodes and the optimization of their ability to reduce protons to molecular hydrogen [95]. Therefore, transition metal compounds including carbides, nitrides, phosphides, and sulfides have been actively investigated for acidic HER. Their higher HER activities than those of pure transition metals can be attributed to the suitable energies for hydrogen adsorption on the catalyst surface [93]. On the other side, the insufficient intrinsic activity of non-noble metals could be improved by tuning properties such as the composition and morphology of the used materials [97]. Non-metallic elements (C, N, P, S, and Se) that are being used in the composites together with metallic elements have high electronegativity and therefore draw electrons from the metal components, as well as attract protons due to the partial negative charge [98].

Although the performances of such materials are still lower than those with noble cathodes, due to the relatively low price of non-noble metals, there is still great scientific interest for research within these alternatives. Incorporation of high-performance low-cost HER electrocatalysts into PEM electrolyzers is an emerging area of research with still limited reports of PEM electrolyzers that utilize non-precious catalysts at the H_2_ electrode side being published [99]. Most of the published works still only deal with the investigation of electrocatalytic activity of novel materials in acidic media by the application of three-electrode systems without further application in PEM water electrolyzers. Both of the mentioned applications will be presented in further sections.

Since the works of Hinnemann et al. [100] and Thomas et al. [101], who reported promising results of the HER electrolysis utilizing MoS_2_, transition metal chalcogenides are among the most promising non-noble metal cathode electrocatalysts. The main characteristic of this family of materials is that they have a 2D lattice structure and, like graphene, the reactive sites are located along the edges while the basal plane is catalytically inactive [7]. Therefore, the aim of most of the works within this area is to increase the number of active sites by increasing the ratio of edges to the basal plane and to increase the electrical conductivity simultaneously while minimizing the overpotential required for HER [7]. For this purpose, many distinct molybdenum sulfide structures such as nanoparticles, nanowires, films, and mesopores were synthesized with the aim of maximizing the number of exposed edge sites [99].

Besides the aspect of active sites, electric conductivity is another crucial factor related to electrocatalytic activity because a high conductivity ensures fast electron transport during the catalytic process [102]. Because of that, MoS_2_, which is more economical and 10^4^ times more abundant than Pt, can be chemically bonded to RGO via a facile solvothermal approach [103]. Such a composite that contains highly exposed edges can exhibit HER activity with a small overpotential of ~0.1 V, large cathodic currents, and a Tafel slope of 41 mV/decade. Another research work conducted by Corrales- Sánchez et al. [104] explored the electrocatalytic activity of MoS_2_/RGO hybrids, as well as of pristine MoS_2_ and MoS_2_ physically mixed with an electrically conducting carbon material (Vulcan^®^ XC72) towards PEM electrolysis. As a reference, the performance of Pt black was also shown. Among tested MoS_2_-based materials, 47 wt% MoS_2_/Vulcan^®^ gave the best performance in terms of current density at an encouraging level for practical application, while the MoS_2_/RGO hybrids showed higher HER activity than pristine MoS_2_. The poor performance of pristine MoS_2_ can be contributed to poor electrical conductivity. Although improvement in electrocatalytic activity for the mentioned composites can be noticed, results still did not exceed those achieved with the use of Pt black.

Research conducted by Kumar et al. [102] confirmed that by controlling the reaction temperature and sulfur precursor employed, different MoS_2_ nanostructures like nanosheets, nanocapsules, and nanoflakes could be obtained. Among all indicated materials, MoS_2_ in the form of nanocapsules exhibits superior activity towards HER in 0.5 M H_2_SO_4_ with an overpotential of 120 mV vs. RHE. The following Mo-based catalyst was further incorporated into the PEM electrolyzer where the fabricated MEA consisted of a Nafion PEM sandwiched between iridium (IV) oxide and MoS_2_-nanocapsules used as the anode and cathode catalyst. The designed cell was operated for 200 h at 2 V without any degradation of electrocatalytic activity.

Recently, another efficient and stable electrocatalyst composed of earth-abundant TiO_2_ nanorods decorated with MoS_2_ thin nanosheets was recorded [105]. This composite possesses hydrogen evolution activity in acidic media at an overpotential of 0.35 V and a Tafel slope of 48 mV/decade. It is very important to measure the Tafel slope because it is a primary and inherent property of the catalyst which indicates the rate-determining step involved in the HER [105]. In this case, the measured Tafel slope is very close to the one of benchmarking Pt/C (32 mV/decade) and indicates that electrochemical desorption is the rate-limiting step for the HER in acidic media.

Higher HER activity than MoS_2_ can be obtained by the use of alternative Mo-based catalysts such as MoS_x_, [MoS_3_S_13_]^2−^ nanoclusters, and sulfur-doped molybdenum phosphide (MoP|S), loaded onto CB support. These carbon-supported catalysts, synthesized by Ng et al. [99], are electrochemically tested in a standard three-electrode electrochemical and subsequently integrated into PEM electrolyzer systems and operated continuously for 24 h. Related to PEM electrolyzer testing, the performance of each electrolyzer was examined by stepping the potential from 1.2 to 1.0 V at 50 mV intervals at a cell temperature of 80 °C. The MoS_x_-CB-based electrolyzer required 1.86 ± 0.03 V to reach 0.5 A cm^−2^, while the MoS_3_S_13_-CB and MoP|S-CB-based electrolyzers both required 1.81 ± 0.03 V to reach 0.5 A cm^−2^. The best overall performance, also including three-electrode electrochemical data, was achieved with the (MoP|S) electrolyzer. Such results suggest that Mo-based catalysts hold promise for commercial applications with the possibility of replacing the Pt-based cathodes currently being used in PEM electrolyzers.

Other interesting transition metal chalcogenides applied as a cathode side within the field of PEMWE are iron sulfide materials, which have the great advantage of being widespread in nature. Pyrite (FeS_2_) is the most abundant sulfide mineral, while pyrrhotite is an unusual iron sulfide mineral with variable iron content [Fe_(1−x)_S_(x=0–0.2)_] that often accompanies base metal sulfides in ore deposits [106,107]. The synthesis, characterization, and activity towards the HER of different stoichiometries of iron sulfide materials including the above-mentioned pyrite and pyrrhotite, as well as greigite (Fe_3_S_4_), were investigated by Di Giovanni et al. [108]. Finally, their performances were also investigated in situ in a PEM electrolyzer single cell under 80 °C. The MEAs were prepared by using pyrite, pyrrhotite, or greigite as the cathode catalyst and tested in an electrolysis single cell. The catalysts were not supported but were mixed with 20% of CB. Nafion 115 (125 µm) was used as the membrane and IrO_2_ as the anode. According to the SEM results presented within the research, the thickness of the IrO_2_ catalyst layer is ~6 µm, while the thickness of the FeS_2_/CB catalyst layer is ~30–40 µm. Also, the experimental results have shown that all three catalysts allow ~2100 mV at 1 A cm^−2^ to be reached, but both ex situ and in situ electrochemical experiments have revealed that pyrite (FeS_2_) is more active than greigite (Fe_3_S_4_), which is more active than pyrrhotite (Fe_9_S_10_). Generally, all three catalysts allow ~2100 mV at 1 A cm^−2^ to be reached.

The electronic structure of metal sulfide materials can be modified by doping metal atoms, which can optimize hydrogen adsorption energy and enhance HER catalytic activity. Such an example can be seen in the work of Wang et al. [109], where Co-doped iron pyrite FeS_2_ nanosheets were hybridized with carbon nanotubes (Fe_1−x_Co_x_S_2_/CNT). HER was tested in 0.5 M H_2_SO_4_ acidic solution in a three-electrode system without further application in PEMWE. Electrochemical measurements showed a low overpotential of ~0.12 V at 20 mA cm^−2^, a Tafel slope of ~46 mV/decade, and long-term durability over 40 h of operation using bulk quantities of Fe_0.9_Co_0.1_S_2_/CNT hybrid catalysts at high loadings (~7 mg cm^−2^). Density functional theory (DFT) revealed that an increase in the catalytic activity comes from a large reduction in the kinetic energy barrier of H atom adsorption on FeS_2_ surface upon Co doping in the FeS_2_ structure.

Transition metal phosphides, such as CoP, NiP, FeP, and MoP, are viewed as a promising replacement of Pt because of their good stability and high activity in acidic media, and further improvement of intrinsic HER activity can be realized by employing more than one transition metal [110]. Therefore, FeCoP shows a near optimal hydrogen adsorption free energy (ΔG_H_) that is similar to that of Pt and is significantly affected by the Fe/Co ratio [110]. The development of NiP catalysts is also on the ascending path, with the composites being developed by different research groups. NiP catalysts electrodeposited on carbon support were developed by Kim et al. [98] and applied as a cathode for a PEMW electrolyzer. The performance of the water electrolyzer was evaluated in a galvanostatic mode in the range of 0.02–4 A cm^−2^ after the activation process, and cell voltages of 1.96, 2.07, and 2.16 were required to obtain a current density of 1.2 and 3 A cm^−2^. NiP nanoparticles, but with a different stoichiometric ration (Ni_2_P) and support (multiwall carbon nanotubes), were designed by in situ thermal decomposition of nickel acetylacetonate as the nickel source and trioctylphosphine as the phosphorus source in an oleylamine solution of carbon nanotubes [111]. Electrocatalytic activity of this nanohybrid was evaluated in 0.5 M H_2_SO_4_ with an onset overpotential of 88 mV, a Tafel slope of 53 mV/decade, and an exchange current density of 0.0537 mA cm^−2^.

Besides nickel–phosphide materials, nickel–carbon-based catalysts were developed by Fan et al. [112]. This work reveals the new area of tuning structure and functionality of metal–carbon-based catalysts at an atomic scale that may help accelerate the large-scale application of PEM electrolyzers. By the use of electrochemical methods, the indicated composite can be activated to obtain isolated nickel atoms anchored on graphitized carbon, consequently displaying high activity and durability for HER. Owing to their low-coordination and unsaturated atoms, isolated metal atoms have demonstrated more catalytic active than nanometer-sized metal particles. Other attempts at the improvement of the “noble-metal-free” electrocatalysts can be noticed at the use of group VI transition metal carbides that exhibit catalytic properties analogous to platinum group materials (PGMs) because of their unique d-band electronic structures [113]. Catalytic properties of carbide materials strongly depend on their surface structure and composition, which are closely associated with their method of synthesis [113]. Therefore, by the use of a simple and environmentally friendly carburization process, Chen et al. [113] synthesized Mo_2_C covalently anchored to carbon supports (carbon nanotubes and XC-72R carbon black). The electrochemical impedance spectroscopy (EIS) results demonstrated that the incorporation of Mo_2_C onto carbon supports enhanced the exchange current density (measured overpotential of 63 mV applied for driving 1 mA cm^−2^ of exchange current density), reduced charge-transfer resistance, and a change in the HER mechanism.

Excellent chemical stability has opened the possibility of the application of transition metal oxides, such as WO_2_ in the field of clean hydrogen energy production. Metallic WO_2_–C mesoporous nanowires with a high concentration of oxygen vacancies (OVs) were synthesized by Wu et al. [114]. All tests were carried out in 0.5 M H_2_-saturated H_2_SO_4_, and the products exhibited promising performance for hydrogen generation with a Tafel slope of 46 mV per decade. For comparison, as already noted, corresponding to the literature [7], the value of the Tafel slope for commercial Pt/C was about 30 mV per decade. Other interesting Pt-free alternatives with the corresponding features are listed in Table 7.

**Table 7:** Non-noble cathode materials for the use in electrolytic acidic hydrogen generation and PEMWE systems.

Cathode Catalyst	Electrochemical Characterization	Membrane	T	Performance	Ref.
MoO_3_ nanowires	Three-electrode cell with 1 M H_2_SO_4_ electrolyte	-	-	11.3 and 56.8 mA cm^−2^ at potential of 0.0 and 0.1 V with a Tafel slope of 116 mV/decade	[115]
Co-Cu alloys	PEMWE single cell with Co-Cu deposited on a carbon paper (CP) as a cathode and IrO_2_ electrodeposited on a CP as an anode	N212 (DuPont)	90 °C	1.2 A cm^−2^ at 2.0 V_cell_	[116]
CoP	Three-electrode cell with 0.5 M H_2_SO_4_ electrolyte	-	-	Current density of 20 mA cm^−2^ at an overpotential of 85 mV	[117]
CoP/CC	Three-electrode cell with 0.5 M H_2_SO_4_ electrolyte	-	-	Onset overpotential of 38 mV with a Tafel slope of 51 mV/decade	[118]
WC@NC	PEMWE single cell with WC@NC as the cathode and IrO_2_ (Sunlaite) as an anode	N212 (DuPont)	80 °C	0.78 A cm^−2^ at 2.0 V_cell_	[119]
OsP_2_@NPC	Three-electrode cell with 0.5 M H_2_SO_4_ electrolyte	-	-	10 mA cm^−2^ at onset overpotential of 46 mV	[120]
NiMo/CF/CP	PEMWE single cell with NiMo/CF/CP as the cathode and IrO_2_/CP as an anode	N212 (DuPont)	90 °C	~2.0 A cm^−2^ at 2.0 V_cell_	[121]
Ni–Mo–N	Three-electrode cell with 0.5 M H_2_SO_4_ electrolyte	-	-	Overpotential of 53 mV at 20 mA cm^−2^	[122]
NiS_2_	Three-electrode cell with 0.5 M H_2_SO_4_ electrolyte	-	-	Overpotential of 213 mV at 10 mA cm^−2^	[123]
NiSe_2_				Overpotential of 156 mV at 10 mA cm^−2^	
NiTe_2_				Overpotential of 276 mV at 10 mA cm^−2^	
MoP/C (NaCl)	Home-made electrolyzer using MoP/C (NaCl) as cathode and IrO_2_ (Sunlaite) as an anode	N211 (DuPont)	80 °C	0.71 A cm^−2^ at 2.0 V_cell_	[124]
MoP@PC	Three-electrode cell with 0.5 M H_2_SO_4_ electrolyte	-	-	Overpotential of 258 mV at 10 mA cm^−2^, with a Tafel slope of 59.3 mV/decade	[125]
MoP@PC	Three-electrode cell with 0.5 M H_2_SO_4_ electrolyte	-	-	Overpotential of 51 mV at 10 mA cm^−2^ with a Tafel slope of 45 mV/decade	[126]
MoP@PC	Three-electrode cell with 0.5 M H_2_SO_4_ electrolyte	-	-	Onset overpotential of 77 mV, overpotential of 153 mV at 10 mA cm^−2^, with a Tafel slope of 66 mV/decade	[127]
MoP/NG	Three-electrode cell with 0.5 M H_2_SO_4_ electrolyte	-	-	Overpotential of 94 mV at 10 mA cm^−2^ with a Tafel slope of 50.1 mV/decade	[128]
MoP/NC	Three-electrode cell with 0.5 M H_2_SO_4_ electrolyte	-	-	Overpotential of 120 mV at 10 mA cm^−2^	[129]
MoP|S	Three-electrode cell with 0.5 M H_2_SO_4_ electrolyte	-	-	Overpotential of 86 mV at 10 mA cm^−2^	[130]
N–Mo_2_C	Three-electrode cell with 0.5 M H_2_SO_4_ electrolyte	-	-	Onset overpotential of 78.1 mV for HER and a Tafel slope of 59.6 mV/decade	[131]
Mo_2_C/C	Three-electrode cell with 0.5 M H_2_SO_4_ electrolyte	-	-	Tafel slope of 56 mV/decade	[132]
Mo_2_C/C	Three-electrode cell with 0.5 M H_2_SO_4_ electrolyte	-	-	Overpotential of 180 mV at 10 mA cm^−2^	[133]
Cu_x_Mo_100−x_/CP	PEMWE single cell with Cu_93.7_Mo_6.3_/CP as the cathode and IrO_2_/CP as an anode	N212 (DuPont)	90 °C	0.50 A cm^−2^ at 1.9 V_cell_	[96]
Cu_1−x_Ni_x_WO_4_	Three-electrode cell with 1 M H_2_SO_4_ electrolyte	-	-	4.3 mA cm^−2^ at the anodic peak potential of 0.09 V	[134]
Ni–P supported by copper foam (CF) on CP	PEMWE single cell with Ni–P/CF/CP as the cathode and IrO_2_/CP as an anode	N212 (DuPont)	90 °C	0.67 A cm^−2^ at 2.0 V_cell_	[135]
NiMo/CF/CP	PEMWE single cell with Ni–Mo/CF/CP as the cathode and IrO_2_/CP as an anode	N212 (DuPont)	90 °C	2.0 A cm^−2^ at 2.0 V_cell_	[121]
FeCo/N–G	Three-electrode cell with 1 M H_2_SO_4_ electrolyte	-	-	Onset overpotential of 88 mV and overpotential of 262 mV at 10 mA cm^−2^	[136]
P–Ag@NC	Three-electrode cell with 1 M H_2_SO_4_ electrolyte	-	-	Overpotential of 78 mV at 10 mA cm^−2^	[137]
Co@N–CNTs@RGO	Three-electrode cell with 0.5 M H_2_SO_4_ electrolyte	-	-	Overpotential of 87 mV at 10 mA cm^−2^	[138]

### 4.2. HER Electrocatalysts with Reduced Noble Metals Content

The last section of this review paper will contribute to electrocatalyst cathode materials which are designed using a smaller proportion of noble metals compared to conventionally used ones. To achieve an environmentally sustainable society, the reduction in the consumption of noble metals is a topic of great importance. In the section above, substituted noble metals alternatives are shown; even though much scientific effort has been put to develop new and prosperous materials, their activities are rarely comparable to that of the benchmark catalyst Pt/C, and their application is still limited in real energy devices. Also, a lot of developed materials are only being tested in acidic media, without further tests in PEMWE systems, which should be another step forward to fully understand their potential use. For these reasons, strategies to synthesize catalysts have mostly been focused on the reduction in the content of noble metals, which is considered a more practical strategy for accelerating their industrial application [139]. Considering commercial PEMWE systems and their industrial applications, the typical catalyst loading is 1–2 mg cm^−2^; therefore, it is responsible for 25% of the PEMWE stack cost [140,141].

To reduce Pt content, different approaches are listed. Some of the solutions imply the development of novel thin and tunable gas diffusion electrodes with a Pt catalyst thickness of 15 nm and a total thickness of about 25 µm, which can enhance catalyst mass activity up to 58 times higher than conventional catalyst-coated membrane (CCM) at 1.6 V under the operating conditions of 80 °C and 1 atm. [142] On the other hand, core-shell structures with Pt on the surface and Ru forming the core of the particles were developed by Ayers et al. [140]. This composition enables appropriate electrocatalytic activity to be achieved; by utilizing Pt spontaneous deposition on metallic Ru nanoparticles, an ultralow Pt-content catalyst was made with a 20:1 Ru:Pt atomic ratio. Furthermore, reactive spray deposition technology (RSDT) enabled one-step fabrication of two MEAs (86 cm^2^) containing platinum group metal (PGM) loadings in amounts of only 0.2 and 0.3 mg_PGM_ cm^−2^ loading in the cathode and anode electrodes, respectively. This assembly, involved in electrolysis operation conducted at 50 °C and 400 psi differential pressure with 1.8 A cm^−2^ current, demonstrated durability for over 3000 h of operation at industrially relevant operating conditions [143].

Consumption of energy to produce hydrogen strongly depends on the current and voltage applied. The cell voltage is composed of the anode and cathode potentials and the *IR* drop in the electrolyte. The reduction in the cathode potential can be achieved by modifying the composition of the catalyst or by increasing the surface area of the catalyst by reducing the particle size [144]. Such an example can be seen in the work of Ravichandran et al. [144] where, during the impregnation reduction synthesis of Pt catalyst, the addition of nonionic surfactant reduced the particle size. The MEAs of 4 cm^2^ coating area, with a loading of 0.4 mg cm^−2^ Pt and 1.2 mg cm^−2^ IrO_2_, was tested for HER and operated as a single cell at 2 V and 80 °C, achieving the highest current density of 1.5 A cm^−2^. Similarly, the stack of the hydrogen generation capacity of about 1 N m^3^ h^−1^ capacity was assembled and tested by the integration of five single MEAs of 500 cm^2^ area. The stack displayed a current density of about 1.18 A cm^−2^ at 10.0 V and 80 °C; performance lasted up to 3000 h of operation with not much change in the current density noticed. The use of the impregnation reduction method of synthesis and surfactant are beneficial in reducing the particle size, which also confirmed earlier conducted studies [145,146]. Wang et al. [145] designed surfactant-stabilized Pt and Pt alloy electrocatalysts on carbon supports for the application in PEMFC and revealed the improved electrocatalytic activity due to the well dispersed and smaller catalytic particles; while Rajalakshmi et al. [146], through the impregnation reduction method, synthesized Pt-deposited Nafion^®^ membrane as cathode without using any surfactant. The obtained material showed improved fuel cell performance in comparison to other methods of synthesis.

To create cathode formulations with cheaper characteristics than Pt, efforts are being addressed to develop Pd-, Rh-, and Ru-based catalysts. Pd is three times less expensive than Pt and was used in the work of Kumar et al. [94] to prepare a phosphorus-doped carbon-nanoparticles-supported palladium (Pd/P-CNPs) electrocatalyst. The structural modification of the carbon by phosphorus doping will be more effective than nitrogen doping since phosphorus has a much larger covalent radius (107 ± 3 pm) than carbon (73 ± 1 pm) compared with nitrogen (71 ± 1 pm). The synthesized electrocatalyst was used as the HER electrode for the fabrication of MEAs, and its performance was evaluated in house-fabricated PEMWE 25 cm^2^ single-cell assemblies. The obtained results showed that the synthesized Pd/P-CNPs have shown similar electrochemical activity and stability compared to commercial Pt/C.

Rh–P catalysts exhibit very good HER performances due to the introduction of P into the Rh catalyst material, which induces a ΔG_H*_ shift to more neutral values; this indicates greater active catalytic activity for a lower amount of Rh loading [97]. Facile fabrication of Rh and Rh–P electrodes on a carbon paper as substrate via electrodeposition at room temperature and ambient pressure was performed by Kim et al. [97] and evaluated for the acidic HER in terms of intrinsic and mass activity. Under the optimized deposition parameters, such as potential and time, a certain facet widespread at the surface of Rh electrodes (Rh (111) facet) demonstrated high intrinsic activity for HER in acidic medial, while the further enhancement of the catalyst performance was achieved by a modified electronic structure of Rh–P electrodes with intrinsic and mass activity greater than ones of Pt electrodes. Except for Rh–P, composites with different stoichiometric ratios of Rh and P are also highly represented within this field. Many different morphologies of Rh_2_P are synthesized and tested for hydrogen production in acidic media with the potential of use in PEMWE. Yang et al. [147] performed a colloidal synthesis of monodisperse Rh_2_P nanoparticles with an average size of 2.8 nm and with an overpotential of 140 mV achieved a current density of 10 mA cm^−2^ in 0.5 M H_2_SO_4_, while Duan et al. [148] synthesized rhodium phosphide nanocubes supported on high surface area carbon (Rh_2_P/NCs). In the case of Rh_2_P/C, the overpotential at the current density of 5 mA cm^−2^ is 5.4 mV, which is lower than Pt/C (8.0 mV) and Rh/C (68.4 mV). Carbon support was also used in the synthesis of wrinkled, ultrathin Rh_2_P nanosheets (w-Rh_2_P NS/C) for enhancing HER in 0.1 M HClO_4_ [149]. To reach a current density of 10 mA cm^−2^, the overpotential of 15.8 mV is required, which is 6.3 and 25.8 mV lower than those of commercial Pt/C (22.1 mV) and carbon-supported Rh nanosheets (Rh NS/C) (41.6 mV). The Tafel slope is 29.9 mV s^−1^, which is comparable for commercial Pt/C and lower than that of Rh NS/C (37.4 mV/decade).

The core-shell structure of obtaining composites used in hydrogen production is well known, and such morphology is also presented in the work of Pu et al. [150], who synthesized Rh_2_P nanoparticles encapsulated in an N-doped carbon (NC) core-shell structure (Rh_2_P@NC) achieving overpotential of 9 mV at 10 mA cm^−2^ in 0.5 M H_2_SO_4_. Comparison in the electrocatalytic activity between Rh_2_P@NC, Rh/NC, and Pt/C and belonging HER polarization curves shows that both Rh_2_P@NC and Pt/C exhibit high HER catalytic activities with 0 mV onset overpotential (*η*_onset_), which is much smaller than that of Rh/NC. Rh_2_P@NC needs an overpotential (*η*_10_) of 9 mV at the current density of 10 mA cm^−2^ with the corresponding Tafel slope of 26 mV/decade. The last part of the electrochemical measurements examined stability test where, after 1000 cyclic voltametric (CV) cycles at a scan rate of 100 mV/s in 0.5 M H_2_SO_4_ solution, the polarization curve retains an almost similar performance to the initial test.

Besides N-doping, carbon-supported materials can also be *double-codoped* with nitrogen and phosphorus. Two electrocatalysts composed of N and P codoped carbon (NPC) modified with noble metal phosphides (Rh_x_P/NPC and RuP/NPC) with a low loading of Rh (≈0.4 wt%) and Ru (≈0.5 wt%) achieved promising electrocatalytic activities [139]. Rh_x_P/NPC delivers Pt-like HER activity with an ultralow overpotential at 10 mA cm^−2^ (19 mV) and a small Tafel slope (36 mV/decade), while the RuP/NPC requires overpotential of 125 mV to achieve 10 mA cm^−2^ and a Tafel slope of 107 mV/decade. Besides metal phosphides, conductive oxides can also be considered as potential catalysts for the HER. The advantage of such materials is that, unlike their metallic counterparts and most prominently Pt, they are not prone to poisoning by underpotential deposition of less active metals that are always presented in the form of impurities in technological electrolytes [151]. Ru and Ir thin films, as well as their corresponding thermally oxidized RuO_2_ and IrO_2_ thin films, were developed by Cherevko et al. [151] and evaluated for HER in 0.1 M H_2_SO_4_. Metals, exhibit more extensive dissolution are found to be more active in catalyzing the hydrogen production, while metal oxides are easily blocked by hydrogen bubbles and show no dissolution during HER. Based on the results, it can be concluded that oxides may be considered to catalyze HER in case Pt contamination is an issue; even though metals are more active, their application as cathode materials is not feasible due to low stability. The dissolution of metals in acidic solutions is 2–3 magnitudes higher compared to their respective oxides.

Table 8 contains selected cathode materials recently synthesized and tested under acidic conditions with a high potential for later application in PEM water electrolysis, according to listed electrochemical parameters (overpotential at current density, Tafel slope, and stability) that are very close to the benchmark 20% Pt/C cathode catalyst material.

**Table 8:** Cathode materials with reduced noble metals content for use in acidic electrolytic hydrogen generation.

Cathode Catalyst	Electrolyte	Overpotential@Current Density	Tafel Slope	Stability	Ref.
Au@AuIr_2_ (core-shell structure nanoparticles (NPs) with Au core and AuIr_2_ alloy shell)	0.5 M H_2_SO_4_	29 mV@ of 10 mA cm^−2^	15.6 mV/decade	40 h	[152]
PdCu/Ir core shell nanocrystals	0.5 M H_2_SO_4_	20 mV@ of 10 mA cm^−2^ same overpotential as a commercial Pt/C	-	15 h@20 mA cm^−2^	[153]
IrPdPtRhRu high entropy alloy (HEA) NPs	0.05 M H_2_SO_4_	33 mV@ of 10 mA cm^−2^ much lower overpotentials to achieve a 10 mA cm^−2^ than the monometallic Ru (77.1 mV), Rh (58.6 mV), Pd (78.4 mV), Ir (47.8 mV) and Pt (48.9 mV)	-	CV for 3000 cycles	[154]
PtRu@RFCs (Pt is alloyed with Ru and embedded in porous resorcinol-formaldehyde carbon spheres) Pt loading 99.9% less than commercial Pt-based catalyst	0.5 M H_2_SO_4_	19.7 mV@10 mA cm^−2^ 43.1 mV @ 100 mA cm^−2^	27.2 mV/decade for comparison: Pt/C (commercial) = 29.9 mV/decade	CV for 5000 cycles	[155]
RuP synthesized by dry chemistry method	0.1 M HClO_4_	36 mV@10 mA cm^−2^ benchmark Pt/C catalyst (20 wt%, Johnson Matthey) = 21 mA cm^−2^	39.8 ± 0.5 mV/decade	CV for 8000 cycles	[156]
Pd_4_S-SNC (palladium sulfide supported by S, N-doped carbon NPs)	0.5 M H_2_SO_4_	32 mV@ of 10 mA cm^−2^	52 mV/decade	CV for 1000 cycles	[157]
PtN_x_ cluster loaded on a TiO_2_ support (PtN_x_/TiO_2_)	0.5 M H_2_SO_4_	67 mV@ of 10 mA cm^−2^	52 mV/decade	CV for 5000 cycles	[158]
Pt nanoclusters (NCs) anchored on porous TiO_2_ nanosheets with rich oxygen vacancies (V_o_-rich Pt/TiO_2_)	0.5 M H_2_SO_4_	-	34 mV/decade much smaller than the Tafel slope of commercial 20% Pt/C (116 mV/decade)	CV for 1000 cycles	[159]
Pt/OLC (onion-like nanospheres on carbon (OLC) with atomically dispersed Pt) 0.27 wt% of Pt	0.5 M H_2_SO	38 mV@10 mA cm^−2^	36 mV/decade for comparison: Pt/C (commercial, 20 wt% of Pt) = 35 mV/decade	100 h@10 mA cm^−2^	[160]

## 5. Challenges and Insights for Future Clean Hydrogen Production Using PEM Water Electrolyzers

Hydrogen production by water electrolysis using electricity from renewable energy sources (solar energy, wind energy, etc.), although still insufficiently represented compared to the production of hydrogen produced from carbon-based sources, has experienced great progress in recent years, especially in the area of research and development.

This is due to the accelerated development of the hydrogen economy, aimed at achieving the targets set in various action plans and strategies at the EU level. In order to achieve the very ambitious EU targets, the future production of electrolyzers must be increased, which must be accompanied by cost reductions and efficiency improvements. Some of the conditions that must be fulfilled are (i) longevity of hydrogen electrolyzers, taking into account all the individual components from which they are composed: in particular, the durability of the electrode material; (ii) the ability to perform well-performing safety measurements during operation and monitoring; and (iii) the ability to quickly detect a potential performance problem and its solution.

In the short to medium term, electrolytically produced hydrogen is expected to find use in certain industries, such as semiconductors and food, where small quantities of high-purity hydrogen are needed to perform basic processes.

Focusing only on hydrogen production by PEM water electrolysis, it is of extreme importance to reduce the noble metal content, especially the platinum content, by at least an order of magnitude and replace it with non-noble metal alternatives in the future. The most promising alternatives are based on transition metals such as tungsten and molybdenum in the form of carbides, phosphides, and sulfides. Another important parameter is the need to increase the electrode area. This could be achieved by implementation of various HER catalysts with different morphological characteristics. A review of the literature shows that changes in the morphological structure of HER electrocatalysts, e.g., core-shell structures, have a positive effect on the increase in the active surface area of the catalytic material itself, but are not sufficient for a wide industrial use. Extensive scientific investigations are urgently needed to address these shortcomings. Furthermore, the role of the cathode material substrate surface is extremely important, and it must assist in the flow of electrons to the current collectors. Doping of carbon supports, which are mostly used as supports for the cathode electrocatalysts, can make them electrochemically active, enable them to have better interaction with the material they are supporting, and also participate in the charge transfer reactions.

All indications are that scientific collaboration is essential if hydrogen is to become the most important energy vector of the present. The planned energy goals for the widespread use of hydrogen will hardly be achieved with an individual approach. The solution with the highest probability of realization is a complementary approach, combining the proposals of leading experts in the field of electrocatalysis, physics, polymer chemistry, environmental chemistry, etc., and therefore, find the best solution with high practical application. Only in this way will PEM electrolyzers become a competitive technology that can be more easily deployed on a large scale.

## 6. Conclusions

Water electrolysis is a promising, renewable technique that enables applications that require small volumes of high purity hydrogen. Among the different types of this technique, of particular importance is PEM water electrolysis due to the dynamic range, reliability, and lack of corrosive electrolyte in comparison with alkaline electrolysis. Wider use at the megawatt scale is limited with the cost of catalyst-coated membrane. Pt supported on carbon black is a conventionally used cathode material that exhibits the best catalytic performance within this field. Since Pt is an extremely expensive noble metal, the increase in PEMWE application requires the development of cheap and long-lived hydrogen evolution reaction electrocatalysts that could substitute the use of Pt-based ones. The most promising non-noble metal alternatives are compounds based on transition metals in the form of sulfides, phosphides, and carbides. Even though much scientific effort is being put in the development of novel materials that do not contain noble metals in their structure, most of the obtained cathode materials still do not exceed the performances of Pt-based electrocatalysts. For these reasons, another part of the scientific community is committed to the development of composites consisting of a lower amount of platinum group materials in combination with other non-noble metal alternatives. Such Pd-, Ru-, Rh-based electrodes are highly efficient in hydrogen production with the potential of large-scale application. But still, further work is needed to improve the activity and stability of the mentioned catalyst, specifically within the context of industrial application. Integration of synthesized catalysts into commercial devices represents an important step forward towards sustainable hydrogen production through proton exchange membrane water electrolysis.

## Figures and Tables

**Table 1:** Current operational state of PEM water electrolyzers and the predicted goals for 2024 and 2030. Modified from [14].

No.	Parameter	Unit	SoA	Targets	
			2020	2024	2030
**1**	Electricity consumption @ nominal capacity	kWh/kg	55	52	48
**2**	Capital cost	EUR/(kg/d) EUR/kW	2100 900	1550 700	1000 500
**3**	O&M cost	EUR/(kg/d)/y	41	30	21
**4**	Hot idle ramp time	s	2	1	1
**5**	Cold start ramp time	s	30	10	10
**6**	Degradation	%/1000 h	0.19	0.15	0.12
**7**	Current density	A/cm^2^	2.2	2.4	3
**8**	Use of critical raw materials as catalysts	mg/W	2.5	1.25	0.25

**Table 2:** List of main companies involved in the manufacturing of PEM water electroyzer systems in Europe. Modified from [22].

Company	Manufacturing Site	Electrolyzer Type
**AREVA H_2_**	France, Germany	PEM
**CarboTech**	Germany	PEM
**Cummins—Hydrogenics**	Belgium, Canada, Germany	PEM and ALKALINE
**DeNora**	Italy, Japan, USA	PEM and ALKALINE
**iGas**	Germany	PEM
**ITM**	UK	PEM
**Nel Hydrogen**	Denmark, Norway, USA	PEM and ALKALINE
**Siemens Energy**	Germany	PEM

**Table 3:** Comparison of water electrolysis technologies. Modified from [22,29].

Comparison between Technologies			
	AWE	PEMWE	SOWE
**Operating temperature**	70–90 °C	50–80 °C	700–850 °C
**Operating pressure**	1–30 bar	<70 bar	1 bar
**Electrolyte**	Potassium hydroxide (KOH) 5–7 mol L^−1^	PFSA membranes	Yttria-stabilized zirconia (YSZ)
**Separator**	ZrO_2_ stabilized with PPS mesh	Solid electrolyte (above)	Solid electrolyte (above)
**Electrode/catalyst (oxygen side)**	Nickel-coated perforated stainless steel	Iridium oxide	Perovskite-type (e.g., LSCF, LSM)
**Electrode/catalyst (hydrogen side)**	Nickel-coated perforated stainless steel	Platinum nanoparticles on carbon black	Ni/YSZ
**Porous transport layer anode**	Nickel mesh (not always present)	Platinum-coated sintered porous titanium	Coarse nickel mesh or foam
**Porous transport layer cathode**	Nickel mesh	Sintered porous titanium or carbon cloth	None
**Bipolar plate anode**	Nickel-coated stainless steel	Platinum-coated titanium	None
**Bipolar plate cathode**	Nickel-coated stainless steel	Gold-coated titanium	Cobalt-coated stainless steel
**Frames and sealing**	PSU, PTFE, EPDM	PTFE, PSU, ETFE	PTFE, silicon
**PEMWE vs. AWE**			
**Advantages**		**Disadvantages**	
*Compact system design* fast heat-up and cool-off time, short response time; low gas cross-permeation; withstands higher operating pressures across the membrane; higher purity of hydrogen and higher thermodynamic voltage; easier hydrogen compression facilitates hydrogen storage. *Solid, thin electrolyte* shorter proton transport route, lower ohmic loss; operates under wide range of power input. *Operation at higher current density* lower operational costs; differential pressure across the electrolyte; pressurized hydrogen side alone (avoidance of danger related to pressurized oxygen).		*Acidic electrolyte* higher manufacturing cost due to expensive materials and components; limited choices of stable earth abundant electrocatalysts for the oxygen evolution reaction (OER). *Solid, thin electrolyte* it can be easily damaged by inappropriate operation (e.g., overheating) and cell design; sensitive to imperfections, dust, and impurities.	

**Table 4:** Commercially available PFSA membranes.

	Manufacturer	Structure	Parameters	Ref.
Nafion^®^	DuPont (Wilmington, DE, USA)	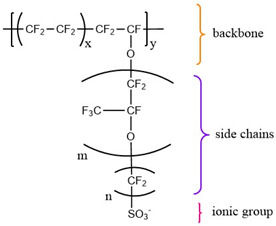	m = 1; n = 2; x = 5–13.5; y = 1000 e.g., EW * = 1000; x = ~5.5	[50,53]
Flemion^®^	Asahi Glass (Tokyo, Japan)		m = 0 or 1; n = 1–5	[53,54,55]
3M^®^	3M™ Corporation (Maplewood, MN, USA)		m = 0; n = 4; x = ~3–5 for EW = 660–825; e.g., for EW = 1000; x = ~6.5 or EW = 700; x = ~3	[50,53,56]
Aciplex^®^	Asahi Kasei (Tokyo, Japan)		M = 0–3; n = 2–5; x = 1.5–14 e.g., for EW = 1130; x = ~7	[53,54,55,57]
Aquivion^®^/Dow SSC^®^	Solvay Specialty Polymers (Brussels, Belgium)		m = 0; n = 2; x = 3.6–10 e.g., for EW = 1000; x = ~7	[50,53]

* EW stands for equivalent weight, grams of dry polymer per ionic group [g mol^−1^].

**Table 6:** Target performance indicators of PEMWE related to advances in membrane materials and catalysts. Modified from [77].

	2022	Target 2050	Research and Development
Nominal current density	1–3 A cm^−2^	4–6 A cm^−2^	Membranes
Voltage	1.4–2.3 V	<1.7 V	Catalysts, membranes
Operating temperature	50–80 °C	80 °C	Durability of the membranes
Cell pressure	≤50 bar	>70 bar	Membranes, catalysts
Load Range	5–130%	5–300%	Membranes
H_2_ purity	99.9–99.9999%	99.9–99.9999%	Membranes
Voltage efficiency (LHV)	50–68%	>80%	Catalysts
Electrical efficiency (stack)	44–66 kWh/kg H_2_	<42 kWh/kg H_2_	Catalysts, membranes
Lifetime (stack)	50,000–80,000 h	100,000–120,000 h	Catalysts, membranes

## Data Availability

Not applicable.
